# 2250 Barriers to healthcare after the Affordable Care Act: A qualitative study of Los Angeles safety net patients’ experiences with insurance and healthcare

**DOI:** 10.1017/cts.2018.238

**Published:** 2018-11-21

**Authors:** Sonali Saluja, Danny McCormick, Michael Cousineau, Janina Morrison, Michael Hochman

## Abstract

OBJECTIVES/SPECIFIC AIMS: N/A. METHODS/STUDY POPULATION: Over a million people gained insurance in Los Angeles (LA) County under the Affordable Care Act (ACA). The vast majority gained Medicaid—government sponsored insurance with low-cost sharing. LA County also made significant investments in the safety net including a program called MyHealthLA, which provides primary and tertiary care for the residually uninsured including poor undocumented individuals at specific sites. Despite this insurance expansion, approximately 3 quarters of a million people in the county remain uninsured. Regardless of insurance status, nearly a quarter of LA County residents reported having difficulty obtaining needed medical care, and among those making less than the poverty level, 43% had difficulties. There is still much to understand about barriers to obtaining insurance and accessing healthcare in Los Angeles in the post-ACA era. Our primary objective was to understand how safety net patients are obtaining, maintaining and using their insurance after the ACA. Specifically we hope to understand the barriers and drivers of these three processes. RESULTS/ANTICIPATED RESULTS: We conducted a qualitative study of 34 safety net patients with 3 different insurance types in LA County. We conducted in-person interviews with adult patients (ages 18–64 years), who had either MediCal, MyHealthLA, or were unsinsured. Our interview guide was based on existing literature, a previous qualitative study conducted in Massachusetts and input from experts in the field. We pilot tested our interviews in English and Spanish and then recruited our participants from 3 sites: LAC+USC (a publically funded county hospital), The Wellness Center (a resource center for safety net patients), and White Memorial Medical Center (a private safety net hospital). We approached patients in the ED and urgent care waiting rooms and obtained informed consent for this IRB approved study. We excluded patients who were non-English and non-Spanish speaking or too ill to interview. We recorded interviews, which were then transcribed and translated into English by a contracted agency. We analyzed our interviews using a framework approach, which included a set of a priori codes from the literature as well as emerging codes from patient responses. We will check a sample of our transcripts for coding consistency (aiming for an inter-rater reliability of >80%). DISCUSSION/SIGNIFICANCE OF IMPACT: We recruited a diverse group of patients that were demographically representative of those who gained insurance under the ACA (childless adults making less than 138% of the Federal Poverty Level). Our preliminary results (based on 17 transcripts), suggest that patients, regardless of insurance type have difficulty accessing primary care. We identified seven domains under the broader theme of barriers to accessing primary care: finding a primary care clinic or physician (PCP), getting timely appointments, geography and transportation, continuity of care, using the Emergency Department (ED) or urgent care as a PCP, switching PCPs or clinics, and cost or coverage.

